# Age influences serum immune indices and gut microbiota composition in adult broilers

**DOI:** 10.3389/fmicb.2026.1802596

**Published:** 2026-06-03

**Authors:** Jialei Chen, Zhuxiang Xiong, Chunlin Yu, Jiangxian Wang, Chunlian Yu, Shiliang Zhu, Han Peng, Li Yang, Chenming Hu, Xia Xiong, Mohan Qiu, Zengrong Zhang, Bo Xia, Xiaoyan Song, Longhuan Du, Chaowu Yang

**Affiliations:** 1 Animal Breeding and Genetics Key Laboratory of Sichuan Province, Sichuan Animal Science Academy, Chengdu, China; 2 The Open University of Sichuan, Chengdu, China

**Keywords:** 16S rDNA sequencing, broiler aging, gut microbiota, host–microbe interaction, serum immune indices, sex difference

## Abstract

While the gut microbiota is recognized as a key modulator of host immunity, its age-related dynamics in adult-to-senescent broilers remain poorly characterized. This study aimed to comprehensively analyze age- and sex-related variations in serum immune indices and gut microbiota composition in yellow-feathered broilers aged 1 to 4 years (*n =* 40). Peripheral blood and fecal samples were gathered for immune profiling using ELISA (STP, IgA, IgG, IL-4, IFN-*γ*) and 16S rDNA sequencing. The results indicated that serum STP levels peaked at 1 year (*p* < 0.01), whereas IgG levels exhibited an age-related increase (*p* < 0.05). Although no significant compositional separation was detected among age groups by PERMANOVA (Adonis *p* = 0.14), within-group beta dispersion differed significantly across ages (*p* < 0.05). Firmicutes and Proteobacteria dominated the fecal microbiota at the phylum level. Exploratory Spearman correlation analysis (based on nominal *p*-values) identified potential associations, such as a positive nominal correlation between Limosilactobacillus abundance and IgG levels (*p* < 0.01), and a negative nominal correlation between Achromobacter and IL-4 (p < 0.01); however, these findings require further validation due to the lack of correction for multiple testing. Notably, one-year-old hens exhibited the highest abundance of beneficial taxa, such as *Lactobacillus*, which corresponded with their elevated STP levels. These findings highlight age- and sex-specific interactions between the gut microbiota and immune responses. From a theoretical perspective, one-year-old hens display a gut microbiota and immune profile that could be favorable for microbiota transplantation; however, direct functional validation (e.g., FMT experiments in recipient models) is required to support this hypothesis. This study offers novel insights into avian aging models and microbiota-mediated immune regulation, presenting potential strategies to enhance poultry health in intensive farming systems.

## Introduction

Broiler chickens, characterized by their short growth cycles and high feed conversion rates, are a vital component of the global livestock industry and play a key role in meeting the human demand for animal protein (Gu et al., 2024). In modern intensive farming models, the industry primarily emphasizes broilers’ growth rate, feed conversion rate, and disease prevention and control, resulting in a significant research gap regarding the later adult and senescent stages of broilers. A comprehensive examination of the factors influencing the growth and development of broilers is crucial for optimizing breeding strategies, improving breeding outcomes, and ensuring the quality and safety of poultry meat. The gut microbiota of broilers is affected by various factors such as environment, breed, age, and sex (Clavijo and Flórez, 2018; Yu et al., 2022). Notably, sex-specific differences in the chicken gut microbiota have been increasingly recognized: female chickens harbor a more stable and complex cecal microbiome than males, with distinct enrichment patterns of specific genera between sexes (Yang et al., 2024). These sex-linked microbiome differences may interact with diet and management interventions in a sex-dependent manner (Leterrier et al., 2025). A healthy and mature gut microbial community is critical for host health, as increased microbial diversity is associated with reduced intestinal disease incidence in poultry (Ocejo et al., 2019). Moreover, the gut microbiota competes with potential pathogens for colonization, stimulates mucosal epithelium and intestinal immune system development, and contributes to nutrient digestion and absorption—serving as a key defense against pathogenic microorganisms (Kubasova et al., 2019). Although an increase in microbial diversity is beneficial to health, the presence of certain microorganisms is also associated with specific health outcomes. For example, the colonization of *Lactobacillus* can promote the weight gain of chicks, while the colonization of *Akkermansia* and *Prevotella* are negatively correlated with weight gain (Zhao et al., 2013). A well - functioning gut microbiota provides many benefits to the host. A rich and highly complex gut microbiota competes with potential pathogens for colonization, helping to exclude pathogenic microorganisms such as *Clostridium perfringens* (Antonissen et al., 2016). In addition, the gut microbiota can also stimulate the development of the mucosal epithelium and intestinal immune system in poultry, providing another way for the host to defend against potential pathogens.

The research results show that as the age increases, the gut microbiota of chickens will evolve from simple to complex and diverse (Awad et al., 2016; Stamilla et al., 2021). In the first few weeks after hatching, the abundance and diversity of intestinal microorganisms in chicks increase with age (Clavijo and Flórez, 2018). As the age increases, the individual differences in the gut microbiota composition of chickens gradually decrease (Kers et al., 2018). As the chicken’s intestine matures, the gut microbiota gradually forms a complex system from a single structure, which plays a very important role in the growth performance and immunity of chickens. Many studies have shown that the gut microbiota is involved in the growth and development, immune regulation, and digestion and absorption of chickens. The gut microbiota of chickens not only affects intestinal development but also has an important regulatory effect on immune defense, growth and development, physiological state, and health. It can be seen that as the age of chickens continues to increase, the composition of their gut microbiota is also constantly changing. The possible mechanism is to increase certain microorganisms with specific functions to better promote the growth and development of chickens.

The stability and diversity of the gut microbiota are important for the host’s health and growth and development. Most existing studies on broiler gut microbiota focus on the growth and development stage (from hatch to market weight, typically 6–8 weeks), while research on adult and senescent broilers (beyond 1 year of age) is extremely limited. Although the temporal development of the gut microbiota has been characterized in commercial layer flocks from hatch to the end of production (Joat et al., 2023) and in native chicken breeds across developmental stages (Wang et al., 2022), these studies primarily address the rearing-to-peak-production phase. Age-related intestinal microbial dysbiosis and chronic low-grade inflammation have been observed in post-peaking laying hens (Hu et al., 2025). However, no studies have systematically examined age-related changes in the gut microbiota of broilers from 1 to 4 years of age, nor their correlation with serum immune parameters. Furthermore, emerging evidence demonstrates that chicken immune cell profiles are significantly influenced by both sex and age, with roosters exhibiting distinct lymphocyte subset compositions and functional capacities compared to hens (Hofmann et al., 2026), yet how these sex-specific immune profiles correlate with gut microbiota composition in adult broilers remains unknown. This study analyzed age- and sex-related differences in the fecal microbiota composition and serum immune indices of adult broilers at different ages. By characterizing the changes in these parameters, we explored the associations between gut microbiota and host physiological/immune status. Our findings provide a descriptive basis for understanding avian aging models and offer a preliminary reference for the theoretical selection of potential donors for fecal microbiota transplantation (FMT). However, direct functional validation, such as FMT experiments in recipient models, is required to test this hypothesis.

## Materials and methods

### Ethics statement

The animal experimental protocol of this project has been reviewed and approved by the Sichuan Academy of Animal Sciences (Permit number: 20210024), and complies with the principles of animal protection and animal welfare, as well as the relevant national regulations on animal welfare ethics (China). This study did not involve any endangered or protected species.

### Experimental design

Fifty 1-day-old Daheng yellow-feathered broilers (both roosters and hens) were selected each year and raised under the same conditions until they were 1 year old. Twenty healthy broilers (both rooster and hen) were randomly selected for further analysis. This cycle was repeated and each batch of chickens was raised under the same conditions. According to different age were divided into the 1-year-old rooster (M1), 1-year-old hen (F1), 2-year-old rooster (M2), 2-year-old hen (F2), 3-year-old rooster (M3), 3-year-old hen (F3), 4-year-old rooster (M4), and 4-year-old hen (F4) groups.

### Sample collection

Peripheral blood (5 mL, blood collection site, brachial vein) was collected from each bird, and the serum was separated (3,000 × g, 10 min, 4 °C) and stored at −20 °C for later use. Two grams of feces was collected from each bird and stored in liquid nitrogen for 16S rDNA sequencing of fecal microorganisms. The sampling quantities and groupings are presented in Table 1.

**Table 1:** Grouping and sample quantity.

Age (years)	Number of roosters	Number of hens	Rooster group identifier	Hen group identifier
1	6	6	M1	F1
2	6	6	M2	F2
3	5	4	M3	F3
4	3	4	M4	F4
Total	20	20	–	–

### Determination of serum physiological and immune indices

The concentrations of serum total proteins (STP), immunoglobulin A (IgA), immunoglobulin G (IgG), interferon-*γ* (IFN-γ), and interleukin-4 (IL-4) were detected using ELISA kits. Experiments were conducted following the manufacturer’s protocols. All ELISA reagents were purchased from Shanghai Enzyme-linked Biotechnology Co., Ltd. (Shanghai, China).

### Extraction of genome DNA and amplicon generation

Total genome DNA from samples was extracted using CTAB method. DNA concentration and purity was monitored on 1% agarose gels. According to the concentration, DNA was diluted to 1 ng/μL using sterile water.

Using diluted genomic DNA as a template, specific primers with barcodes were used according to the selection of the sequencing region. All PCR reactions were carried out with 15 μL of Phusion® High-Fidelity PCR Master Mix (New England Biolabs); 2 μM of forward and reverse primers, and about 10 ng template DNA. Thermal cycling consisted of initial denaturation at 98 °C for 1 min, followed by 30 cycles of denaturation at 98 °C for 10 s, annealing at 50 °C for 30 s, and elongation at 72 °C for 30 s. Finally 72 °C for5min. Primers for the 16S V4 region were 515F (5’-GTGCCAGCMGCCGCGGTAA-3′) and 806 R (5’-GGACTACHVGGGTWTCTAAT-3′).

### Mixing and purification of PCR products

Mix same volume of 1X loading buffer (contained SYBR Green) with PCR products and operate electrophoresis on 2% agarose gel for detection. PCR products was mixed in equidensity ratios. Then, mixture PCR products was purified with Qiagen Gel Extraction Kit (Qiagen, Germany).

### Library preparation and sequencing

Sequencing libraries were generated using TruSeq® DNA PCR-Free Sample Preparation Kit (Illumina, USA) following manufacturer’s recommendations and index codes were added. The library quality was assessed on the Qubit@ 2.0 Fluorometer (Thermo Scientific) and Agilent Bioanalyzer 2,100 system. At last, the library was sequenced on an Illumina NovaSeq platform (NovaSeq 6,000) and 250 bp paired-end reads were generated.

### Bioinformatics and statistical analysis

Paired-end reads were assembled and quality-filtered using fastp (v0.22.0), and sequences were merged with FLASH (v1.2.11) (Magoč and Salzberg, 2011). Chimeric sequences were detected and removed by comparing tags against the SILVA (16S/18S) or Unite (ITS) database using the UCHIME algorithm (Edgar et al., 2011; Haas et al., 2011). Effective sequences were then clustered into operational taxonomic units (OTUs) at 97% sequence identity using UPARSE (v7.0.1001) (Edgar, 2013); alternatively, amplicon sequence variants (ASVs) were resolved using Deblur. Taxonomic assignment for 16S rRNA sequences was performed with the Mothur-based classifier against the SILVA database (Quast et al., 2013). Multiple sequence alignment of representative ASV sequences was conducted with MAFFT (v7.490) (Katoh et al., 2002). ASV abundance tables were rarefied to the minimum library size prior to downstream analysis. Alpha diversity (community richness: Chao1, ACE; community diversity: Shannon, Simpson; sequencing depth: Good’s coverage) was calculated using QIIME and visualized with R software (v4.1.2). Beta diversity was evaluated by weighted and unweighted UniFrac distances, principal component analysis (PCA), principal coordinate analysis (PCoA), and unweighted pair-group method with arithmetic means (UPGMA) hierarchical clustering, the inter-group differences in *β* diversity were tested by PERMANOVA (Adonis), and the intra-group distance dispersion was tested by Kruskal-Wallis test, all implemented in QIIME and R (stats, ggplot2).

The results were expressed as mean ± standard error (SEM). For serum immune indices (STP, IgA, IgG, IL-4, IFN-*γ*): a two-way analysis of variance (two-way ANOVA) was performed to evaluate the main effects of age (1, 2, 3, 4 years), sex (rooster vs. hen), and their interaction. When a significant main effect was detected, Tukey’s honest significant difference (HSD) *post hoc* test was used for multiple comparisons among groups. All two-way ANOVA analyses were conducted using GraphPad Prism v8.0 (GraphPad Software, San Diego, CA, USA). For microbiota data (alpha diversity, beta diversity, Metastats, LEfSe, and KEGG prediction): given the large number of comparisons, the false discovery rate (FDR) Benjamini-Hochberg method was applied to adjust *p*-values. Only results with an adjusted p-value < 0.05 were considered statistically significant for these exploratory analyses.

Functional profiles were inferred using PICRUSt2, which predicts the functional gene content of microbial communities from 16S rRNA gene sequences based on ancestral state reconstruction and hidden state prediction against reference genomes (Langille et al., 2013). After copy number normalization of 16S rRNA operons, metagenomic functional potentials were calculated by multiplying predicted gene family copy numbers per organism by the relative abundance of each ASV. It should be noted that PICRUSt2 predictions are in silico inferences that inherently carry uncertainty, particularly for organisms lacking closely related sequenced reference genomes, and do not directly reflect measured gene expression or metabolic activity.

To identify genus-level taxa driving differences between key pairwise groups, Welch’s t-test was applied to genera with a mean relative abundance > 0.1% in at least 20% of samples within each comparison. *p*-values were adjusted using the Benjamini-Hochberg FDR method, and genera with an FDR-adjusted *p* < 0.05 were considered significantly different. For group pairs where FDR correction yielded no significant results due to limited sample size, nominal p-values are reported for descriptive purposes.

To focus the analysis on robust functional categories, only KEGG Pathway Level 2 results are presented in the main text, while KEGG Level 1 results are provided as Supplementary material. Differential abundance of PICRUSt2-predicted KEGG Level 2 pathways between groups was assessed using Welch’s t-test. To control for multiple testing, *p*-values were adjusted using the Benjamini-Hochberg false discovery rate (FDR) method. Only pathways with an FDR-adjusted p < 0.05 were considered statistically significantly different.

**Figure 1:**
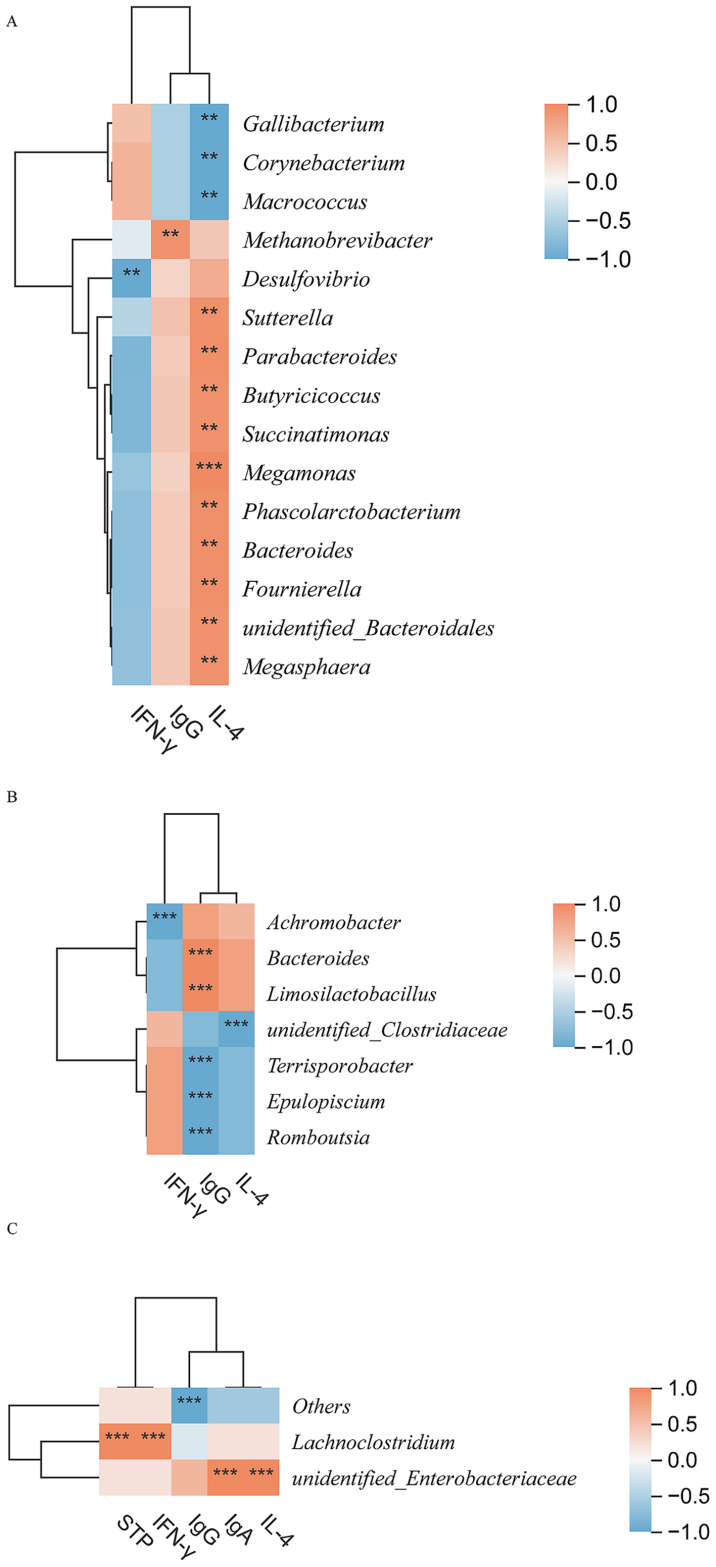
Heat map of correlation analysis between fecal microbiota and serum immune indicators of hen broilers in different ages: **(A)** 1-year group hens; **(B)** 3-year group hens; **(C)** 4-year group hens. The *x*-axis represents bacterial serum immune markers, and the *y*-axis represents genus names.

Spearman’s rank correlation analysis was performed using an online cloud platform to evaluate associations between the relative abundance of bacterial genera and serum immune indices. Due to the functional limitations of the platform, only nominal (uncorrected) *p*-values were available, and the Benjamini-Hochberg false discovery rate (FDR) correction could not be applied. To mitigate the risk of false positives, only correlations with a nominal *p* < 0.01 were highlighted in the heatmaps (Figure 1). Given the exploratory nature of this analysis, these nominal p-values should be interpreted with caution and considered as hypothesis-generating leads for future validation rather than definitive associations. All statistical tests were two-tailed. Raw sequence data of this study have been deposited to the NCBI Sequence Read Archive with accession no. PRJNA1393619.

## Results and analysis

### Determination results of serum physiological and immune indices in adult yellow-feathered broilers of different ages

The detection results of the serum immune indices in adult broilers of different ages are shown in Tables 2, 3, with the complete two-way ANOVA (or linear mixed-effects model) results summarized in Tables 4, 5.

**Table 2:** Results of serum STP, IgA and IgG content of adult broilers at different ages.

Age (years)	STP content (μg/mL)		IgA content (μg/mL)		IgG content (μg/mL)	
	Rooster	Hen	Rooster	Hen	Rooster	Hen
1	2950.32 ± 162.93a	3429.68 ± 30.70a	321.13 ± 5.96a	328.43 ± 3.82a	958.18 ± 77.04c	1028.64 ± 16.01b
2	1174.51 ± 46.25b	1675.12 ± 198.49bc	288.03 ± 4.31b	324.96 ± 1.98a	1274.76 ± 60.38b	1330.91 ± 34.25a
3	1421.09 ± 119.39b	1266.98 ± 33.15c	326.38 ± 8.48a	349.55 ± 20.37a	1357.90 ± 14.29b	1414.23 ± 20.09a
4	1474.22 ± 61.05b	1812.62 ± 170.44b	340.23 ± 5.11a	328.02 ± 8.09a	1571.22 ± 49.62a	1387.61 ± 38.51a

**Table 3:** Results of serum IL-4 and IFN- *γ* content of adult broilers of different ages.

Age (years)	IL-4 content (pg/mL)		IFN-γ content (pg/mL)	
	Rooster	Hen	Rooster	Hen
1	59.07 ± 2.91a	66.55 ± 1.51a	59.60 ± 1.15b	62.27 ± 0.48b
2	61.38 ± 7.95a	61.63 ± 2.23a	76.38 ± 0.82a	76.06 ± 0.41a
3	61.90 ± 1.77a	62.81 ± 2.22a	76.62 ± 1.64a	79.55 ± 2.11a
4	67.12 ± 1.04a	62.82 ± 2.06a	61.92 ± 6.58b	72.75 ± 4.86a

**Table 4:** Supplementary statistical results for IgA, IgG and STP.

Effect	IgA			IgG			STP		
	*F* (DFn, DFd)	*p*-value	Significance	*F* (DFn, DFd)	*P*-value	Significance	*F* (DFn, DFd)	*P-*value	Significance
Age	*F*(1.814, 9.072) = 6.85	0.017	*	*F*(3,5) = 49.4	<0.0001	****	*F*(3, 15) = 104.5	<0.0001	****
Sex	*F*(1, 5) = 6.01	0.058	ns	*F*(1,5) = 0.07961	0.7891	ns	*F*(1, 5) = 8.471	0.0334	*
Age × Sex	*F*(1.516, 3.537) = 3.38	0.151	ns	*F*(3,7) = 3.891	0.0656	ns	*F*(3, 7) = 2.747	0.1223	ns

**Table 5:** Supplementary statistical results for IL-4 and IFN-γ.

Effect	IL-4			IFN-γ		
	*F* (DFn, DFd)	*P-*value	Significance	*F* (DFn, DFd)	*P-*value	Significance
Age	*F*(3, 32) = 0.2247	0.8785	ns	*F*(0.9317, 4.659) = 30.55	0.0035	**
Sex	*F*(1, 32) = 0.1390	0.7117	ns	*F*(1.000, 5.000) = 6.762	0.0482	*
Age × Sex	*F*(3, 32) = 0.7118	0.5521	ns	*F*(0.9922, 2.315) = 2.034	0.2733	ns

For serum total protein (STP), the mixed-effects model revealed a significant main effect of age [*F*(3, 15) = 104.5, *p* < 0.0001] and a significant main effect of sex [*F*(1, 5) = 8.471, *p* = 0.0334], while the age × sex interaction was not significant [*F*(3, 7) = 2.747, *p* = 0.1223; Table 4]. Overall, STP content in the 1-year-old group of broilers was the highest and was significantly higher than that in the 2-year-old, 3-year-old, and 4-year-old groups (*p* < 0.05). There was no significant difference in STP content among the 2-year-old, 3-year-old, and 4-year-old groups (*p* > 0.05). Across all ages, hens exhibited significantly higher STP levels than roosters (main effect of sex, *p* = 0.0334). Although the age × sex interaction was not significant, descriptive comparison of the data suggested a notable trend of higher STP in hens at 1 and 2 years of age, which diminished in older birds; however, these within-age sex comparisons should be interpreted with caution.

For serum IgA, the mixed-effects model showed a significant main effect of age [*F*(1.814, 9.072) = 6.85, *p* = 0.017], whereas the main effect of sex was not significant [*F*(1, 5) = 6.01, *p* = 0.058] and the age × sex interaction was also not significant [*F*(1.516, 3.537) = 3.38, *p* = 0.151; Table 4]. Among all groups, serum IgA levels in the M2 group were significantly lower than those in the M1, M3, M4, and F2 groups (*p* < 0.05), with no significant differences observed among the remaining groups (*p* > 0.05).

For serum IgG, the mixed-effects model revealed a significant main effect of age [*F*(3, 5) = 49.4, *p* < 0.0001], while the main effect of sex [*F*(1, 5) = 0.07961, *p* = 0.7891) and the age × sex interaction [*F*(3, 7) = 3.891, *p* = 0.0656] were not significant (Table 4). Serum IgG content in adult roosters and hens showed a consistent, significant increase with age; IgG content in the 2-year-old, 3-year-old, and 4-year-old groups was significantly higher than that in the 1-year-old group for both roosters and hens (*p* < 0.05).

For serum IL-4, two-way ANOVA indicated no significant main effects of age [*F*(3, 32) = 0.2247, *p* = 0.8785] or sex [*F*(1, 32) = 0.1390, *p* = 0.7117], and no significant age × sex interaction [*F*(3, 32) = 0.7118, *p* = 0.5521; Table 5]. Serum IL-4 content remained stable across all eight groups, with no significant differences detected (all *p* > 0.05), indicating that serum IL-4 content in adult chickens of different ages was stable and did not change systematically with age or sex.

For serum IFN-*γ*, the mixed-effects model showed a significant main effect of age [*F*(0.9317, 4.659) = 30.55, *p* = 0.0035] and a significant main effect of sex [*F*(1, 5) = 6.762, *p* = 0.0482], while the age × sex interaction was not significant [*F*(0.9922, 2.315) = 2.034, *p* = 0.2733; Table 5]. Serum IFN-γ levels in the M2 and M3 groups were significantly higher than those in the M1 and M4 groups (*p* < 0.05), and IFN-γ levels in the F2, F3, and F4 groups were significantly higher than those in the F1 group (*p* < 0.05). Overall, hens exhibited slightly higher IFN-γ levels than roosters (main effect of sex, *p* = 0.0482).

Data were analyzed by two-way ANOVA or linear mixed-effects model. Different uppercase letters in the same column indicate significant differences among age groups (Tukey’s HSD *post hoc* test, *p* < 0.05). In the absence of significant age × sex interactions (see Tables 4, 5), simple main effects of sex within each age group are not compared unless explicitly indicated.

### 16S rDNA sequencing results of fecal bacteria in adult broilers of different ages

#### Quality analysis of sequencing results

**Figure 2:**
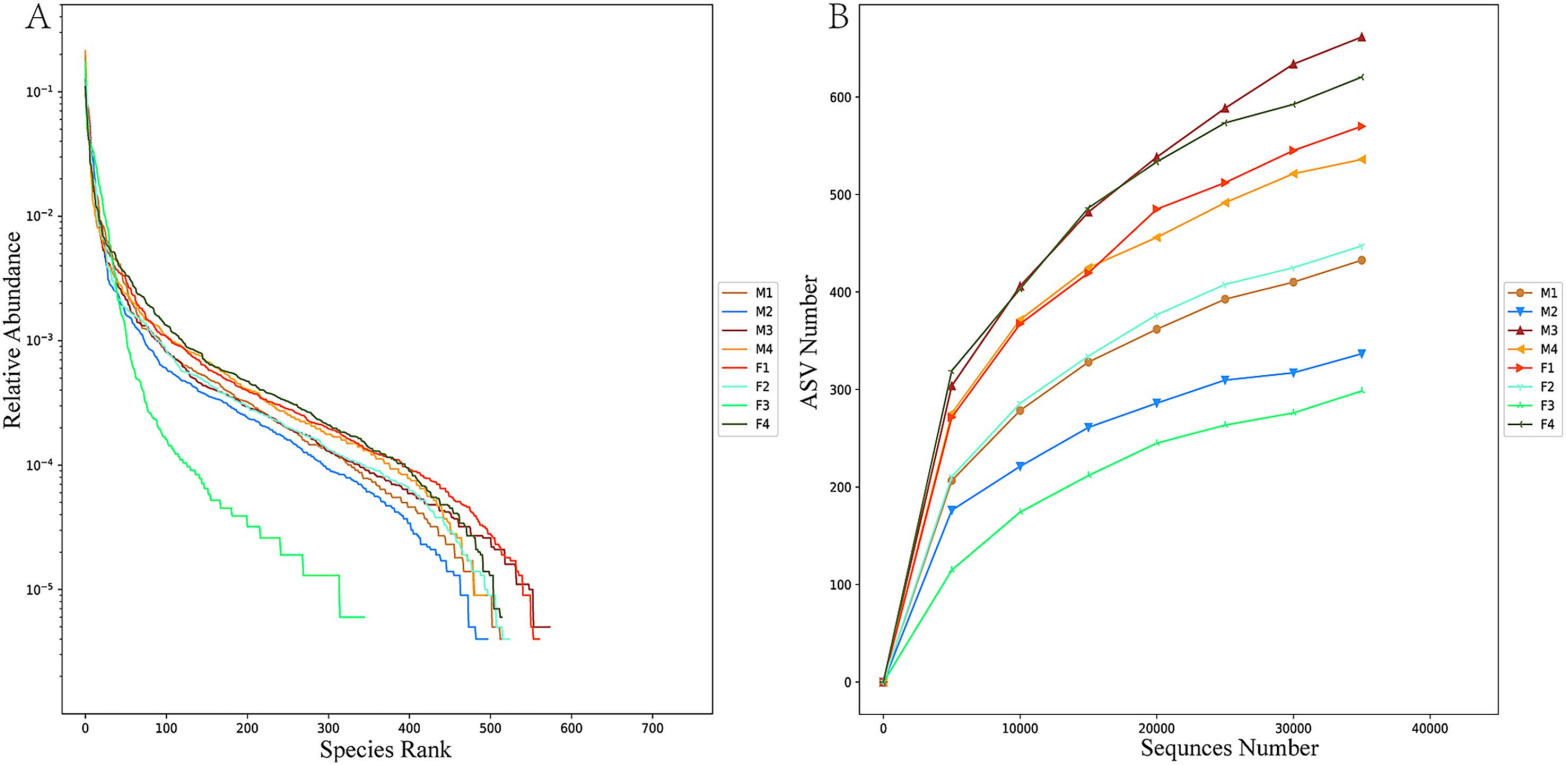
Sample feasibility analysis. **(A)** Rank abundance curves based on amplicon sequence variant (ASVs). In the rank abundance curve, the *x*-axis represents the rank order of ASVs by abundance, and the *y*-axis represents the relative abundance of the corresponding ASVs. Different groups are represented by polylines in different colors. **(B)** Rarefaction curves based on ASVs. In the rarefaction curve, the *x*-axis represents the number of sequencing reads randomly sampled from each group, and the *y*-axis represents the number of ASVs that can be constructed based on that number of reads. This curve reflects the sequencing depth. Different sample groups are represented by curves in different colors. “M1,” “M2,” “M3,” “M4,” “F1,” “F2,” “F3” and “F4” represent the different eight groups, the same applies to the following figures.

Raw data (Raw PE) obtained from Illumina NovaSeq sequencing were quality controlled and spliced to obtain Clean Tags, and chimera filtering was performed to obtain effective data (Effective Tags) for subsequent analysis. As shown in Table 6, the number of sample Clean Tags was between 63,236 and 119,188, and the average length of each sequence was greater than 400 bp, indicating that the integrity and accuracy of the sequencing were reliable. The results are presented in Table 6. As the sequencing depth increased, the rarefaction curves of each group tended to flatten (Figure 2A), indicating that the sequencing depth was sufficient to cover the bacterial species in the samples and meet the requirements for subsequent analysis. The rank abundance curve revealed that the F3 group had lower species richness and evenness compared to other groups (Figure 2B).

**Table 6:** The quantity of chicken fecal sample and average length statistics.

Sample_name	Raw_tags	Clean_tags	Mean_length (bp)
M1_1	103,227	102,831	429
M1_2	103,776	103,357	427
M1_3	102,481	101,559	422
M1_4	105,361	104,971	429
M1_5	119,674	119,188	420
M1_6	104,232	103,845	424
M2_1	116,344	115,873	429
M2_2	115,695	115,289	429
M2_3	104,728	104,336	418
M2_4	104,805	104,368	429
M2_5	105,137	104,715	423
M2_6	102,906	102,476	427
M3_1	105,471	105,060	429
M3_2	117,843	117,400	427
M3_3	115,412	114,936	422
M3_4	111,840	111,437	427
M3_5	105,920	105,448	425
M4_1	91,992	91,652	428
M4_2	102,084	101,704	429
M4_3	101,225	100,788	421
F1_1	104,275	103,891	429
F1_2	115,739	115,306	423
F1_3	107,814	107,436	426
F1_4	105,473	105,072	420
F1_5	102,860	102,471	428
F1_6	105,172	104,747	428
F2_1	103,902	103,520	420
F2_2	102,714	102,256	427
F2_3	102,190	101,728	420
F2_4	80,667	80,293	416
F2_5	108,459	108,040	428
F2_6	104,950	104,526	424
F3_1	104,720	104,353	425
F3_2	103,711	103,336	427
F3_3	105,133	104,640	420
F3_4	103,293	102,835	420
F4_1	63,531	63,236	428
F4_2	118,653	118,140	421
F4_3	104,804	104,414	414
F4_4	102,375	101,894	416

### Sequencing results and alpha diversity analysis of fecal bacterial flora in adult broilers of different ages

**Figure 3:**
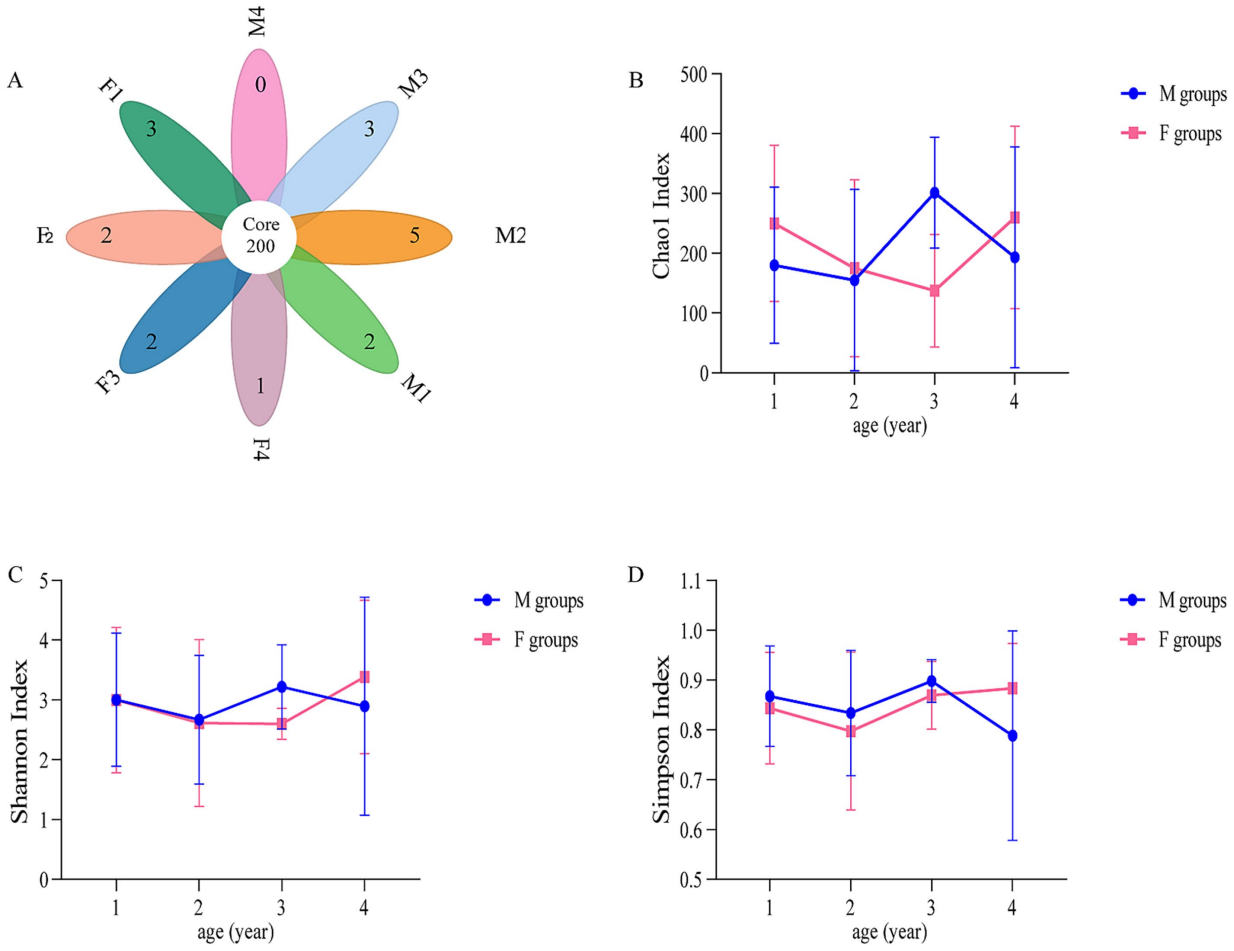
DNA sequence data and alpha diversity index analysis. **(A)** Venn diagram. The numbers in the figure represent the unique or common ASVs in each group. **(B)** Chao1 index (community richness estimator). **(C)** Shannon index (community diversity). **(D)** Simpson index (community evenness).

A total of 217 ASVs were identified in all samples, of which 200 were common to all samples, accounting for approximately 92.7%. In addition, there were two unique ASVs in the M3, F2, and F3 groups, two unique ASVs in the M1 and F1 groups, four unique ASVs in the M2 group, one unique ASV in the F4 group, and no unique ASV in the M4 group (Figure 3A). There were no significant differences in the Chao1, Shannon, and Simpson indices between the groups (*p* > 0.05) (Figures 3B–D).

### Beta diversity analysis of fecal bacterial flora in adult yellow-feathered broilers of different ages

**Figure 4:**
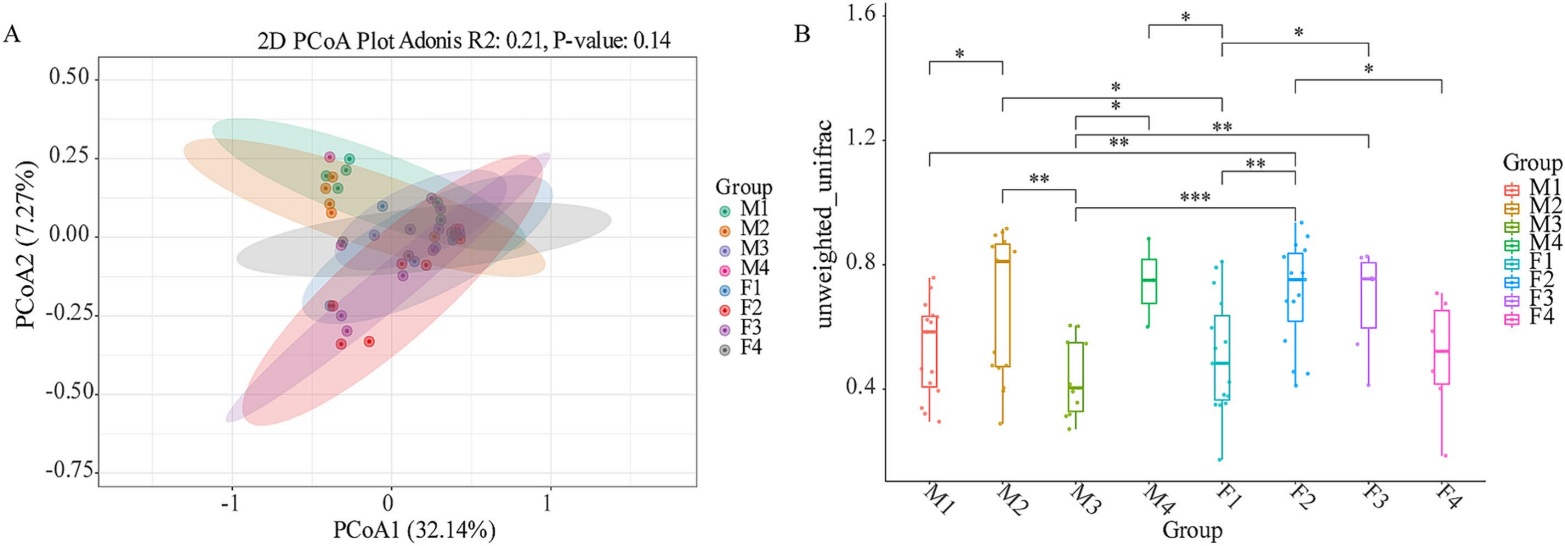
Beta diversity analysis of the fecal bacterial flora of adult yellow-feathered broilers at different ages. **(A)** ASV-based principal component analysis (PCoA) of analysis of unweighted unifrac distance. The *x*-axis represents one principal component, and the *y*-axis represents another principal component. The percentages indicate the contribution of the principal components to the sample differences. Each point in the plot represents a sample, and samples from the same group are shown in the same color. **(B)** Boxplot of unweighted unifrac beta diversity based on ASV (Kruskal-Wallis test). The *x*-axis represents the group names, the *y*-axis represents the within-group unweighted unifrac index, and the horizontal line in the middle of the boxplot represents the median value. “*” Represent the difference was significant *p < 0.05, **p < 0.01, and ***p < 0.001 the same as below.

As shown in Figure 4A, the first two principal coordinates of the PCoA based on unweighted UniFrac distances accounted for 47.07 and 10.7% of the total variance, respectively. A PERMANOVA (Adonis) test indicated no significant overall difference in microbial community composition among the age groups (*p* = 0.14). To further evaluate differences in community heterogeneity, we compared within-group unweighted UniFrac distances (beta dispersion) across groups. The Kruskal-Wallis test revealed significant differences in beta dispersion among the groups (*p* < 0.05; Figure 4B). Specifically, in the same age group, there was a significant difference between the M3 and F3 groups (*p* < 0.01), and there was no significant difference between the different gender groups in the other age groups (*p* > 0.05).

### Composition and difference analysis of fecal bacterial flora in adult broilers of different ages at different taxonomic levels

**Figure 5:**
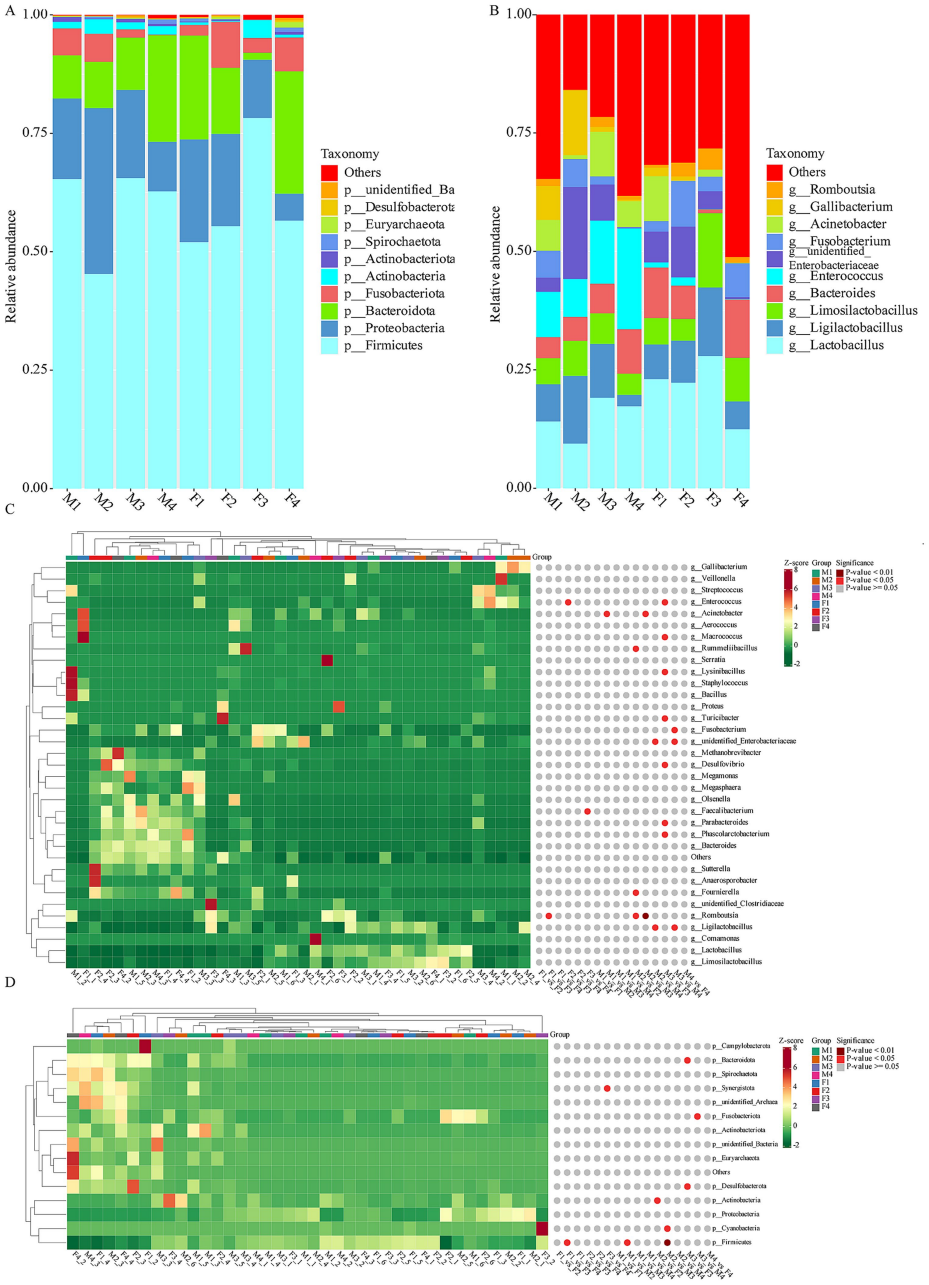
Bacterial community composition and metastat analysis at different taxonomic levels. **(A)** Relative abundance at the phylum level of each group. **(B)** Relative abundance at the genus level of each group. The *x*-axis represents the sample names; the *y*-axis (relative abundance) represents the relative abundance; “Others” represents the sum of the relative abundances of all other phyla (genera) apart from the 10 phyla (genera) shown in the figure. **(C)** Metastats analysis at the genus level of each group. **(D)** Metastats analysis at the phylum level of each group. Different colors on the right indicate the metastats significance of the microorganisms in the corresponding differential grouping.

At the phylum level, the five most abundant phyla are Firmicutes, Proteobacteria, Bacteroidetes, Fusobacteria, and Actinobacteria. The dominant phylum in the feces of adult broilers was Firmicutes, followed by Proteobacteria, and Bacteroidetes. Further details are presented in Figure 5A. The results of the Metastats analysis (Figure 5C) showed that the relative abundance of Firmicutes in the feces of broilers in the M1 group was significantly higher than that in the M2 group (*p* < 0.05), the relative abundance of Firmicutes and Cyanobacteria in the feces of broilers in the M3 group were significantly higher than those in the M2 group (*p* < 0.05), and the relative abundance of Fusobacteria was significantly higher than that in the M4 group (*p* < 0.05). The relative abundance of Firmicutes in the feces of broilers in the F3 group was significantly higher than that in the F1 group (*p* < 0.05), and the relative abundance of Synergistota was significantly lower than that in the F4 group. The relative abundance of Actinobacteria in the feces of broilers in the M2 group was significantly higher than that in the F2 group, and the relative abundances of Bacteroidetes and Desulfobacterota in the feces of broilers in the M3 group were significantly higher than those in the F3 group (*p* < 0.05).

As shown in Figure 5B, the genera with higher abundance in the feces of all broiler groups were *Lactobacillus*, *Ligilactobacillus*, *Limosilactobacillus*, *Bacteroides*, *Enterococcus*, and unidentified Enterobacteriaceae. The Metastats analysis results (Figure 5D) showed that among the dominant genera, there were no significant differences in the relative abundances of *Lactobacillus*, *Limosilactobacillus*, and *Bacteroides* in the feces of broilers in each group (*p* > 0.05), and the relative abundances of *Ligilactobacillus*, *Enterococcus*, and *unidentified Enterobacteriaceae* were significantly different among the different age and sex groups (*p* < 0.05). The relative abundances of some non-dominant genera, such as *Romboutsia*, *Peptococcus*, *Faecalibacterium*, and *Parabacteroides*, also showed significant differences among the groups (*p* < 0.05).

### Analysis of the relative abundance of fecal probiotics, LEfSe analysis and t-test results at the genus level

**Figure 6:**
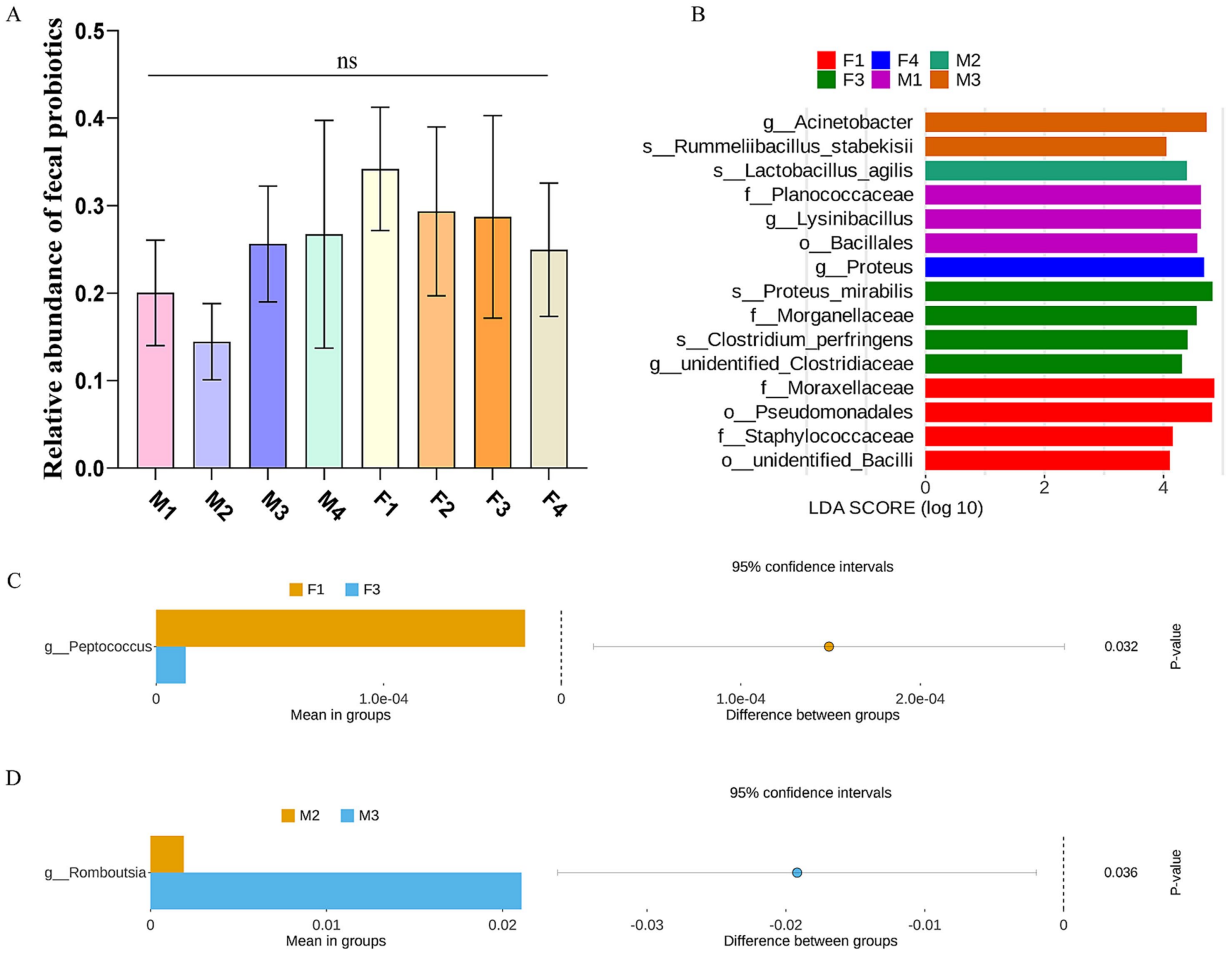
Analysis of relative abundance of fecal probiotics and LEfSe. **(A)** Relative abundance of fecal probiotic bacteria in yellow-feathered broilers at different ages. **(B)** ASV-based bar chart of LDA value distribution. The bar chart of LDA value distribution shows the species with LDA scores greater than the set threshold (default is 4), i.e., the biomarkers with statistically significant differences between groups. It displays species with significant abundance differences among different groups, and the length of the bars represents the effect size of the differential species (i.e., the LDA score). **(C)** The *t*-test results at the genus level for the F1 group versus the F3 group. **(D)** The *t*-test results at the genus level for the M2 group versus the M3 group.

We selected representative and widely studied probiotic genera, including *Lactobacillus*, *Bifidobacterium*, *Bacteroides*, *Bacillus*, and *Lachnoclostridium* as indicators for the evaluation of relative probiotics abundance in each group. Figure 6A shows that the relative abundance of probiotics in the feces of the M2 group was the lowest, while that of the F1 group was the highest. The relative abundance of probiotics in the rooster group reached the lowest value at 2 years of age, whereas in the hen group, it decreased slowly with increasing age. There were no significant differences in the relative abundance of probiotics among the groups (*p* > 0.05).

LEfSe analysis was used to screen for core microbiota markers in the feces of yellow-feathered broilers of different ages. As shown in Figure 6B, 15 core markers were found in the feces of yellow-feathered broilers aged 1–4 years. The M1 group was mainly enriched in Planococcaceae, *Lysinibacillus*, and Bacillales. The M2 group was mainly enriched in *Lactobacillus agilis*. The M3 group was enriched in *Acinetobacter* and *Rummeliibacillus_stabekisii*. The F1 group was mainly enriched in Moraxellaceae, Pseudomonadales, Staphylococcaceae, and unidentified_Bacilli. The F3 group was mainly enriched in *Proteus mirabilis*, Morganellaceae, *Clostridium perfringens*, and *unidentified_Clostridiaceae*. The F4 group was mainly enriched in *Proteus*.

The t-test results showed that the abundance of *Peptococcus* in group F1 was significantly higher than that in group F3 (Figure 6C, *P* = 0.032), and the abundance of *Romboutsia* in group M3 was significantly higher than that in group M2 (Figure 6D, *P* = 0.036); no bacterial genus showed significant differences in the t-test for the other groups.

### KEGG function prediction analysis

**Figure 7:**
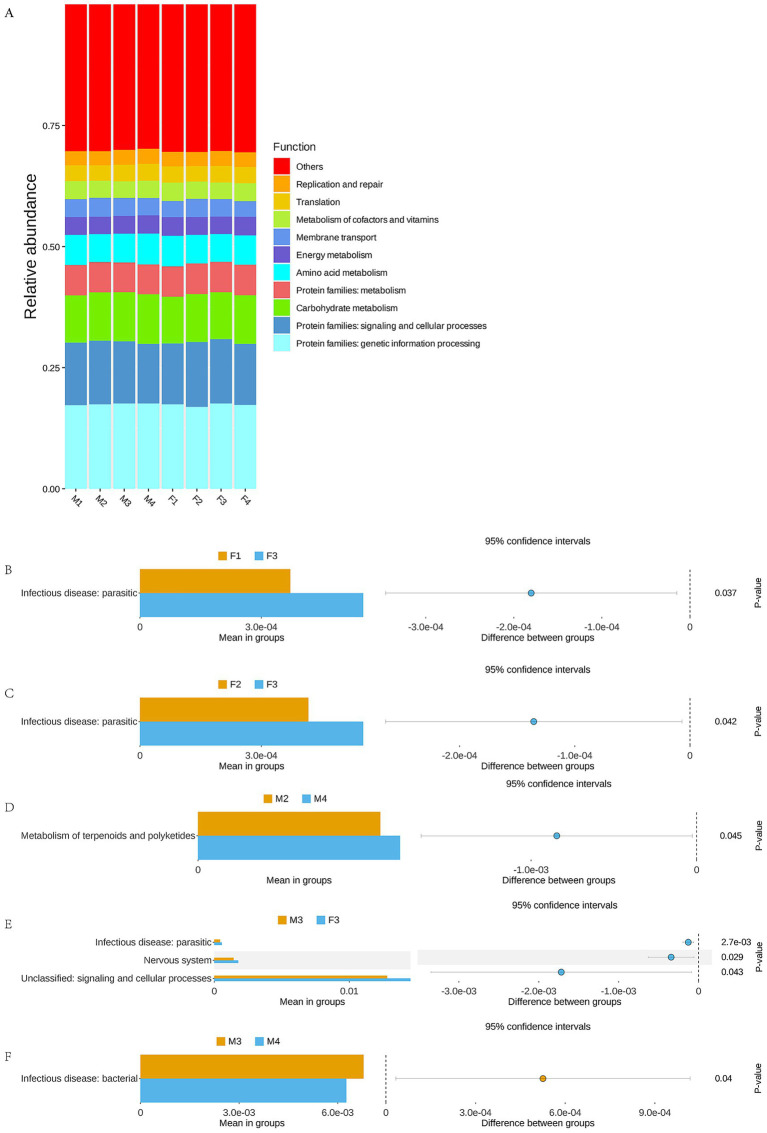
*In silico* functional prediction and differential analysis at KEGG pathway level 2 by PICRUSt2. **(A)** Predicted functional annotation at KEGG level 2. The *x*-axis represents the group names, and the *y*-axis (relative abundance) represents the predicted relative abundance of functional categories. **(B–F)** Differentially predicted pathways between indicated groups (Welch’s *t*-test). Asterisks denote pathways with significant differences after Benjamini-Hochberg FDR correction (*FDR-adjusted *p* < 0.05). These functional inferences represent computationally predicted genomic potential and do not reflect directly measured gene expression or metabolic activity.

The in silico-predicted functional profiles of the fecal microbiota at KEGG Pathway Level 2 are presented in Figure 7. It should be emphasized that these represent computationally inferred genomic potential rather than directly measured gene expression or metabolic activity. As shown in Figure 7A, predicted functional categories with the highest relative abundance across all groups were “protein families: genetic information processing” and “protein families: signaling and cellular processes.” Differential analysis with FDR correction (Figures 7B–F) indicated that the predicted abundance of “infectious diseases: parasitic” in the F3 group was significantly higher than that in the F1 and F2 groups (FDR-adjusted *p* < 0.05). The predicted abundance of “metabolism of terpenoids and polyketides” in the M2 group was significantly higher than that in the M4 group (FDR-adjusted *p* < 0.05). The predicted abundance of “infectious diseases: bacteria” in the M3 group was significantly higher than that in the M4 group (FDR-adjusted *p* < 0.05). Additionally, the predicted abundances of “infectious diseases: parasitic,” “nervous system,” and “unclassified: signaling and cellular processes” in the F3 group were significantly higher than those in the M3 group (FDR-adjusted *p* < 0.05).

### Correlation analysis between Fecal microorganisms and serum immune markers in adult broilers of different ages

Spearman’s rank analysis was used to evaluate the correlations between fecal microbiota composition and serum immune markers (e.g., IgG, IgA, STP) in adult broilers of different ages (1–4 years) using heatmap analysis. The Spearman correlation analysis, based on nominal *p*-values, revealed numerous potential associations between microbial taxa and immune markers with dynamic shifts across age groups (Figure 1). Only correlations reaching a nominal threshold of *p* < 0.01 are highlighted.

**Figure 8:**
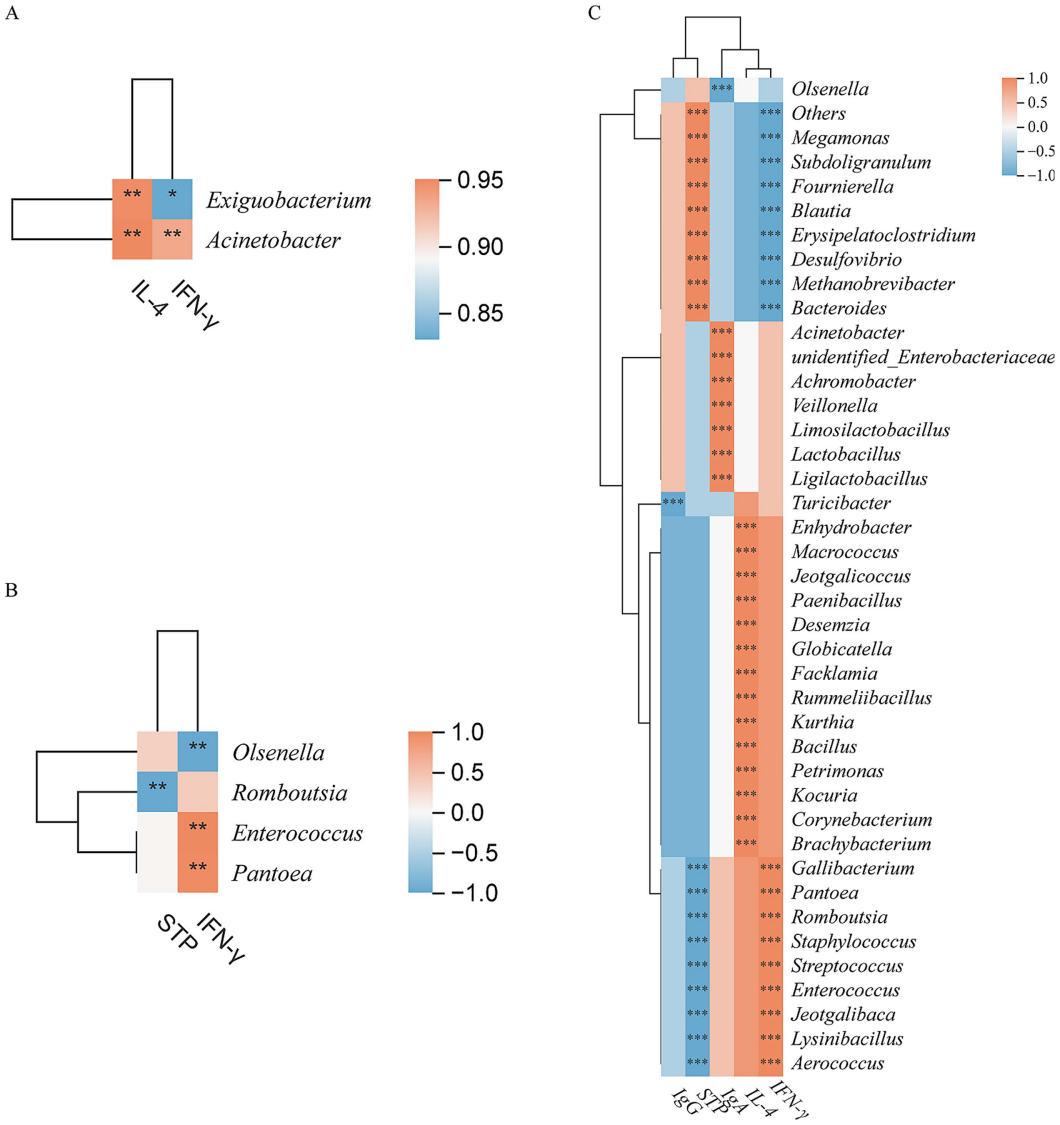
Heat map of correlation analysis between fecal microbiota and serum immune indicators of rooster broilers in different ages. **(A)** 1-year group roosters. **(B)** 2-year group roosters. **(C)** 4-year group roosters. The *x*-axis represents bacterial genus names, and the *y*-axis represents serum immune markers. The strength and significance of associations are based on nominal (uncorrected) *p*-values. Only correlations with a nominal *p* < 0.01 are marked: *p < 0.01, ** *p* < 0.001, *** *p* < 0.0001. These highlighted associations should be considered exploratory and interpreted with caution due to the lack of correction for multiple testing. The same applies to Figure 1.

In the M1 group (Figure 8A), *Acinetobacter* showed a nominal positive correlation with IFN-*γ* and IL-4 (*p* < 0.01), while *Exiguobacterium* displayed a nominal positive correlation with IL-4 and a nominal negative correlation with IFN-γ (*p* < 0.01). In the M3 group (Figure 8B), *Enterococcus* and *Pantoea* exhibited nominal positive correlations with IFN-γ (*p* < 0.01), whereas *Olsenella* showed a nominal negative correlation with IFN-γ (*p* < 0.01); *Romboutsia* was nominally positively correlated with STP. In the M4 group (Figure 8C), genera such as *Megamonas*, *Fournierella*, *Bacteroides*, and *Desulfovibrio* showed nominal positive correlations with STP and nominal negative correlations with IFN-γ (*p* < 0.0001). Conversely, *Gallibacterium*, *Staphylococcus*, and *Enterococcus* exhibited nominal negative correlations with STP and nominal positive correlations with IFN-γ (*p* < 0.0001).

In the F1 group (Figure 1A), *Enterococcus*, *Corynebacterium*, and *Macrococcus* showed nominal negative correlations with IL-4 (*p* < 0.001), while *Megamonas*, *Bacteroides*, and *Faecalibacterium* exhibited nominal positive correlations. *Methanobrevibacter* was nominally positively correlated with IgG (*p* < 0.001), and *Desulfovibrio* showed a nominal negative correlation with IFN-*γ* (*p* < 0.001). In the F3 group (Figure 1B), *Limosilactobacillus* and *Bacteroides* displayed nominal positive correlations with IgG (*p* < 0.0001), whereas *Terrisporobacter*, *Epulopiscium*, and *Romboutsia* exhibited nominal negative correlations with IgG (*p* < 0.0001). In the F4 group (Figure 1C), *Lachnoclostridium* showed nominal positive correlations with STP and IFN-γ (*p* < 0.0001), and *unidentified_Enterobacteriaceae* was nominally positively correlated with IgA and IL-4 (*p* < 0.0001).

## Discussion

In the early growth stage, broilers exhibit rapid growth and active organ/system development, with high nutrient requirements; energy is primarily allocated to muscle growth and bone formation. As they age, especially in the later adult stage, the growth rate slows down, and metabolism shifts toward maintaining basic body functions and coping with aging-related changes. In this study, serum total protein (STP) content peaked at 1 year of age and then significantly decreased. One possible interpretation of this observation is that hepatic synthetic function may decline with age, which would be consistent with the age-related decline in organ metabolic function reported in mammals (David et al., 2014). However, our data do not directly measure liver function, and other factors (e.g., nutritional status, protein metabolism) could also contribute to the observed STP changes. Similarly, it has been reported that the activity of the cytochrome P450 enzyme system in aged broilers is lower than that in young broilers, which might lead to slower metabolism of certain drugs. Notably, serum IgG levels in aged broilers (≥ 3 years) were significantly increased, whereas IgA levels remained relatively stable. This pattern could suggest that systemic humoral immune response is enhanced or maintained with age, while the mucosal immune response remains relatively stable. This phenomenon is in line with the characteristics of humoral immune memory observed in human and mouse studies (Shang et al., 2016; Ma et al., 2024). Regarding cellular immunity, the ratio of Th1/Th2 cytokines (IFN-γ/IL-4) showed that IFN-γ levels increased from 1 to 3 years of age and then declined. This age-related pattern may be interpreted as a potential sign of immunosenescence, i.e., a gradual decline in T cell function with age (Franceschi et al., 2000; Liu et al., 2023). The concept of age-dependent immune decline (immunosenescence) in poultry is further supported by recent evidence showing that post-peaking laying hens exhibit systemic inflammatory responses, oxidative stress, and intestinal barrier dysfunction associated with gut microbiota dysbiosis (Hu et al., 2025), as well as age-related shifts in both mucosal morphology and cytokine expression profiles in the ileum of broilers (Duangnumsawang et al., 2023). The interplay between host-related factors (age, breed, and sex) has been shown to significantly influence bacterial metabolite production and immune traits in the broiler ileum (Duangnumsawang et al., 2023), highlighting the complexity of the age-sex-microbiota-immune axis that our current study begins to disentangle. Nevertheless, our study does not directly measure T cell subsets or telomere length, and the observed cytokine changes could also be influenced by other physiological or environmental factors. Gender differences in immune indicators observed in this study (e.g., numerically higher STP in the F1 group than in the M1 group, consistent with the significant main effect of sex on STP; higher serum IgA in F2 hens than in M2 roosters, though the sex main effect for IgA was not significant) suggest that sex-specific regulatory mechanisms may be at play. It is well established that estrogen enhances immunoglobulin production and promotes B lymphocyte development in the oviduct of chickens (Zheng et al., 1997), while androgen exposure during embryonic development can suppress immune function (Simkins et al., 2020). These endocrine-mediated immune effects may partially explain the generally higher STP levels in hens (significant main effect of sex) and the more favorable immune-microbiota profiles observed in the F1 group. At the microbiota level, a recent integrated 16S rRNA sequencing and metabolomics study demonstrated that the cecal microbiome of female chickens is more stable and complex than that of males, with Lactobacillus enriched in males and Romboutsia and Lachnoclostridium enriched in females (Yang et al., 2024). These sex-specific microbial signatures are consistent with our observation that Romboutsia showed sustained correlations with immune markers predominantly in hens. Additionally, Hofmann et al. (2026) reported that roosters have fewer peripheral B cells and lower antibody concentrations but more γδ T cells compared to hens, suggesting a greater reliance on innate immunity in males (Hofmann et al., 2026). This sex-biased immune strategy could underlie the differential microbiota-immune interaction patterns we observed. Taken together, these findings suggest that sex hormones, via their effects on both the immune system and the gut microbial ecosystem, may jointly shape the sex-specific microbiota-immune interaction patterns observed in adult broilers, an aspect that could be explored in future studies using transcriptomics and hormone profiling.

Numerous studies have shown that age affects the gut microbiota in humans and mammals (Poroyko et al., 2011; Zhao et al., 2015; Wang et al., 2018). Similarly, in broilers, the gut microbiota maintains a close interaction with the host throughout life, and this interaction evolves with age. During the chick stage, colonization of the gut microbiota is a key process, and its composition is relatively simple and unstable (Glendinning et al., 2019). Previous studies have shown that the intestinal microbiota of chicks becomes increasingly rich and stable with age (Bilal et al., 2021; Stamilla et al., 2021) however, in the 1- to 4-year-old adult chickens in this study, there was no significant age difference in the *α*-diversity index, suggesting that the community richness and evenness may have reached a plateau after sexual maturity. In the present study, the dominant bacterial phyla in adult broiler feces were Firmicutes, Proteobacteria, and Bacteroidetes, which is consistent with previous reports (Videnska et al., 2014; Broom and Kogut, 2018). Although α-diversity did not differ significantly among age groups, PERMANOVA (Adonis) analysis based on unweighted UniFrac distances revealed no significant overall compositional separation among the age groups (*p* = 0.14). In contrast, Kruskal-Wallis analysis of within-group UniFrac distances (beta dispersion) indicated significant differences in community heterogeneity across ages (*p* < 0.05). This pattern of stable diversity but compositional shift could be interpreted as an indication that the host’s physiological status (e.g., immunity and metabolism) may exert selective pressure on the microbiota, leading to functional differentiation rather than simply increasing species richness. However, this interpretation remains correlative, and causal relationships cannot be inferred from our data. For instance, the significantly higher abundance of Firmicutes and Cyanobacteria in the M3 group might be associated with enhanced fiber-metabolic capacity, as suggested by previous studies (Thoetkiattikul et al., 2013), while the lower abundance of Synergistota in the F4 group could hypothetically reflect reduced nitrogen metabolic efficiency in aged hens. These speculations require direct metabolic measurements for validation. Notably, the abundance of probiotic genera (e.g., *Lactobacillus*) was highest in the F1 group and positively correlated with serum IgG. One possible explanation, based on the literature, is that microbiota-immune interactions may be mediated by microbial metabolites such as short-chain fatty acids (Koh et al., 2016). Nevertheless, we did not measure metabolite concentrations, and this proposed mechanism remains to be tested in future studies. The lowest abundance of probiotics in the M2 group, along with decreased STP and IgA levels, might point to a stage-specific metabolic or immune stress in young roosters, leading to an imbalance in microbiota-host homeostasis. Further research is needed to explore the functional implications of these correlational findings.

When considering the potential suitability of broilers as donors for FMT, the F1 group (1-year-old hens) exhibited several characteristics that could be theoretically favorable. In terms of serum immune indicators, the F1 group showed relatively high STP content, which may reflect an active physiological state. However, whether such a state would translate into beneficial effects for FMT recipients remains unknown. Regarding gut microbiota, although alpha diversity did not differ significantly among groups, beta diversity analysis revealed that the F1 group had a distinct compositional structure. Additionally, the relative abundances of some dominant phyla and genera (e.g., Firmicutes and *Lactobacillus*) in the F1 group appeared relatively stable. These taxa are generally considered important for intestinal health and immune regulation based on previous literature. Nevertheless, our observational data do not directly demonstrate any functional advantage of the F1 group as FMT donors. Direct validation through FMT experiments in recipient animal models is necessary to test this hypothesis.

In contrast, whether aged broilers (3-year-old group and above) might have different suitability as donors remains unknown and requires direct functional evaluation. However, this recommendation still requires further research and verification, including the evaluation of application effects in different disease models and breeding environments.

This study reveals sex differences in microbiota-immune correlations in adult broilers. In adult rooster broilers, lactic acid bacteria (e.g., *Limosilactobacillus*, *Lactobacillus*) showed consistent positive correlations with immune markers across ages, suggesting their ongoing role in supporting immune function via mechanisms such as maintaining intestinal barrier integrity and modulating inflammatory responses. In contrast, adult hen broilers exhibited broader positive correlations in youth, with notable genus succession (e.g., strengthened correlation of *Romboutsia*) over time, possibly related to reproductive cycles, hormonal fluctuations, and metabolic demands. Sex hormones (e.g., estrogen, androgen) are known to influence gut permeability, immune cell activity, and microbiota composition, which may underlie the observed sex-specific interaction patterns. In addition, we identified several bacterial genera that showed consistent correlations with immune markers, including lactic acid bacteria, *Romboutsia*, and *Achromobacter*. Lactic acid bacteria were positively correlated with immune markers in both sexes, which aligns with their recognized potential as probiotics in poultry. Notably, *Achromobacter*, showed negative correlations in hens across multiple age stages but not in roosters. One possible interpretation is that *Achromobacte*r may play an immunosuppressive or pro-inflammatory role specifically in hens, although this hypothesis is speculative and requires direct experimental testing (e.g., gnotobiotic or *in vitro* models). Similarly, the sustained positive correlation of *Romboutsia* with immune markers in hens could be tentatively linked to its potential production of short-chain fatty acids, which are known immune modulators (Koh et al., 2016). However, we did not measure SCFA concentrations, and the correlational nature of our data does not support a causal link. These observations should be considered hypothesis-generating rather than mechanistic conclusions. Future functional studies, including metagenomics, metabolomics, or microbial transplantation experiments, are needed to clarify the roles of these genera in broiler immunity. In summary, the gut microbiota structure and immune function of adult broilers dynamically changed with age.

While alpha diversity remained relatively stable, the genus-level compositional shifts and in silico-predicted KEGG Level 2 functional potentials suggested possible age-related remodeling of microbial community structure and genomic functional capacity. However, these PICRUSt2 predictions are based solely on 16S rRNA gene sequences and reference genomes; they do not capture actual gene expression, metabolic activity, or the contributions of poorly characterized taxa lacking closely related sequenced genomes. Therefore, these predicted functional differences should be interpreted as hypothesis-generating leads that require validation through shotgun metagenomics, metatranscriptomics, or metabolomics. The core findings of this study are the age- and sex-associated patterns at the genus level, including the higher relative abundance of beneficial taxa such as Lactobacillus and Bacteroides in the F1 group, which corresponded with favorable serum immune parameters. The 1-year-old hens (F1 group) exhibited a combination of relatively high probiotic abundance and active serum immune parameters (e.g., STP and IgG), which may suggest theoretical suitability as donors for fecal microbiota transplantation (FMT). However, this interpretation is based on observational correlations; direct functional validation through FMT experiments in recipient models is required to test this hypothesis. This study provides descriptive data that may contribute to the construction of avian aging models and the development of healthy breeding strategies, although the molecular mechanisms underlying microbiota–immune interactions and their potential applications remain to be systematically explored.

## Conclusion

This study unveils an age-dependent trajectory of the fecal microbiota in adult yellow-feathered broilers that is decoupled from alpha diversity yet tightly linked to functional remodeling and systemic immunity. While microbial richness plateaued after sexual maturity, beta-dispersion and taxonomic signatures shifted progressively, culminating in a senescent configuration characterized by reduced probiotic lineages, shifts in in silico-predicted KEGG functional modules, and a Th1-skewed cytokine profile. Critically, one-year-old hens combined a relatively high abundance of beneficial taxa (e.g., *Lactobacillus*, *Bifidobacterium*, *Bacteroides*, and *Bacillus*) with peak serum total protein and IgG titers. These characteristics suggest that the one-year-old hens may possess a theoretically favorable gut microbiota and immune status for consideration as donors in future FMT studies. Nevertheless, this interpretation remains speculative without direct FMT experiments, and future studies should employ gnotobiotic or antibiotic-treated recipient models to causally link age-specific taxa with immune outcomes. These findings establish the yellow-feathered broiler as a tractable avian model for dissecting microbiota–immune interactions across the adult-to-senescent transition, and provide a data-driven framework for precision microbiota engineering in sustainable poultry production. Future work should couple gnotobiotic transfer with multi-omics readouts to causally link age-specific taxa with immune ontogeny and productive lifespan.

## Data Availability

The original contributions presented in the study are publicly available. This data can be found here: NCBI Sequence Read Archive, BioProject accession number PRJNA1393619.
