# Supplementary figures and images for: Age influences serum immune indices and gut microbiota composition in adult broilers
Source: Front Microbiol. 2026 Jun 3;17:1802596. doi: 10.3389/fmicb.2026.1802596 (PMC13272331; doi:10.3389/fmicb.2026.1802596)

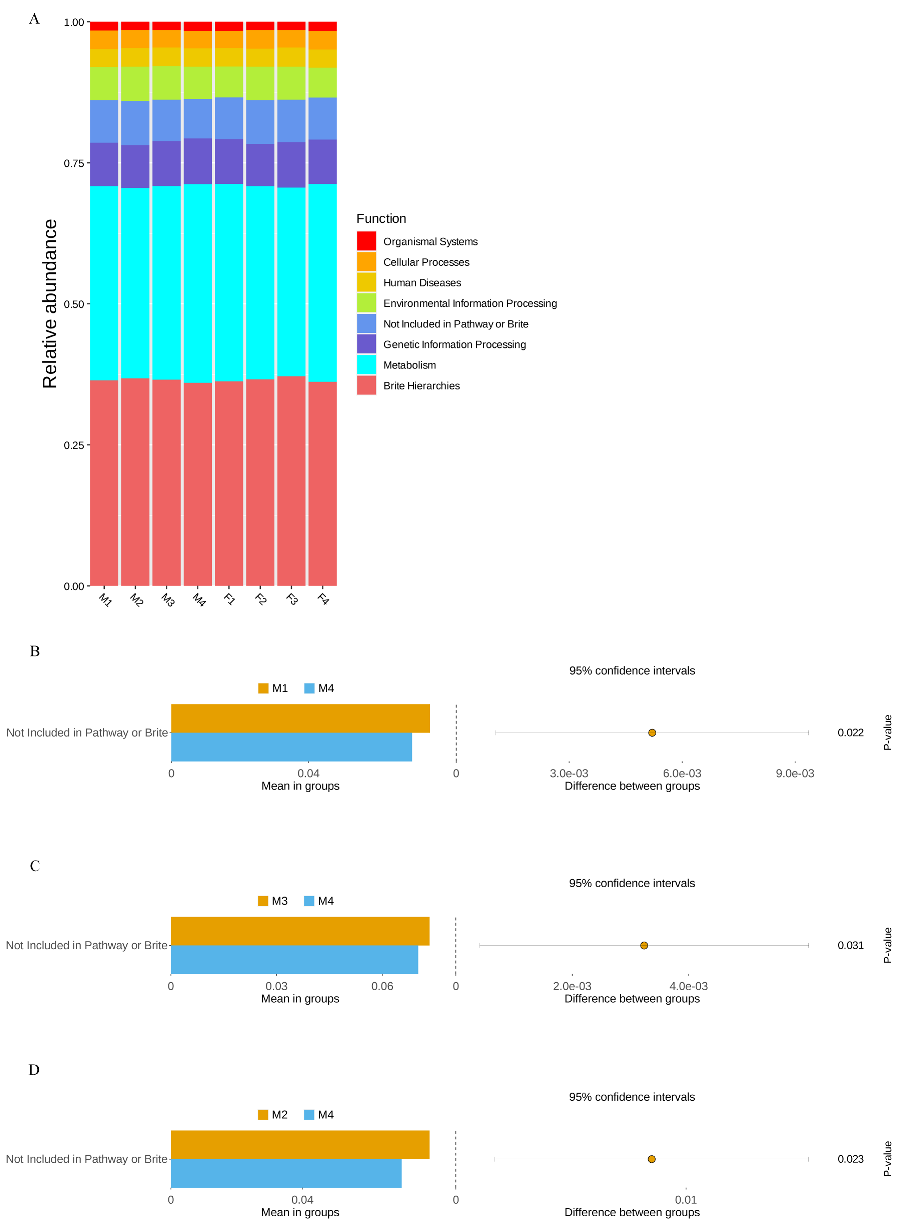

Supplement: Supplementary file 1 [file Image_1.tif]
